# CD8αα Expression Marks Terminally Differentiated Human CD8+ T Cells Expanded in Chronic Viral Infection

**DOI:** 10.3389/fimmu.2013.00223

**Published:** 2013-08-06

**Authors:** L. J. Walker, E. Marrinan, M. Muenchhoff, J. Ferguson, H. Kloverpris, H. Cheroutre, E. Barnes, P. Goulder, Paul Klenerman

**Affiliations:** ^1^Peter Medawar Building for Pathogen Research, Nuffield Department of Medicine, University of Oxford, Oxford, UK; ^2^Institute of Cellular Medicine, Newcastle University, Newcastle upon Tyne, UK; ^3^KwaZulu-Natal Research Institute for Tuberculosis and HIV, K-RITH, Nelson R Mandela School of Medicine, UKZN, Durban, South Africa; ^4^Department of International Health, Immunology and Microbiology, University of Copenhagen, Copenhagen, Denmark; ^5^La Jolla Institute for Allergy and Immunology, San Diego, CA, USA; ^6^National Institute for Health Research Oxford Biomedical Research Centre, Oxford, UK

**Keywords:** CD8+ T cells, hepatitis B, hepatitis C, HIV-1, CD8α

## Abstract

The T cell co-receptor CD8αβ enhances T cell sensitivity to antigen, however studies indicate CD8αα has the converse effect and acts as a co-repressor. Using a combination of Thymic Leukemia (TL) antigen tetramer, which directly binds CD8αα, anti-CD161, and anti-Vα7.2 antibodies we have been able for the first time to clearly define CD8αα expression on human CD8 T cells subsets. In healthy controls CD8αα is most highly expressed by CD161 “bright” (CD161++) mucosal associated invariant T (MAIT) cells, with CD8αα expression highly restricted to the TCR Vα7.2+ cells of this subset. We also identified CD8αα-expressing populations within the CD161 “mid” (CD161+) and “negative” (CD161−) non-MAIT CD8 T cell subsets and show TL-tetramer binding to correlate with expression of CD8β at low levels in the context of maintained CD8α expression (CD8α+CD8β^low^). In addition, we found CD161−CD8α+CD8β^low^ populations to be significantly expanded in the peripheral blood of HIV-1 and hepatitis B (mean of 47 and 40% of CD161− T cells respectively) infected individuals. Such CD8αα expressing T cells are an effector-memory population (CD45RA−, CCR7−, CD62L−) that express markers of activation and maturation (HLA-DR+, CD28−, CD27−, CD57+) and are functionally distinct, expressing greater levels of TNF-α and IFN-γ on stimulation and perforin at rest than their CD8α+CD8β^high^ counterparts. Antigen-specific T cells in HLA-B^∗^4201+HIV-1 infected patients are found within both the CD161−CD8α+CD8β^high^ and CD161−CD8α+CD8β^low^ populations. Overall we have clearly defined CD8αα expressing human T cell subsets using the TL-tetramer, and have demonstrated CD161−CD8α+CD8β^low^ populations, highly expanded in disease settings, to co-express CD8αβ and CD8αα. Co-expression of CD8αα on CD8αβ T cells may impact on their overall function *in vivo* and contribute to the distinctive phenotype of highly differentiated populations in HBV and HIV-1 infection.

## Introduction

CD8α is a cell surface glycoprotein that can be expressed either as a disulfide-linked heterodimer together with CD8β or as a homodimer. In contrast to CD8αβ, CD8αα is never expressed on naïve T cells but readily induced on strongly activated T cells (1). In humans and mice, CD8αα can be expressed on double negative or CD4+ or CD8αβ+ CD3+ TCRαβ+ T cells whereas CD3+ TCRγδ+ T cells and NK cells express mostly CD8αα alone (2).

In mice, CD8αα-expressing populations are the predominant intraepithelial lymphocyte subset of the small bowel and either TCR γδ+ or αβ+ (3). CD8αα has also been identified as a marker of murine memory CD8αβ TCRαβ+ cells, with affinity induced expression in early T cell responses identifying a memory precursor population in both an LCMV (4) and *Listeria* model (1). In humans, we have recently shown single-positive (SP) CD8αα T cells (i.e., those expressing no detectable CD8β) to be exclusively derived post-thymically from a naïve CD161++CD8+ T cell pool with a predominant usage of the Vα7.2 TCR of Mucosal Associated Invariant T (MAIT) cells (5). In addition, CD8αα has recently been shown using the Thymic leukemia (TL) tetramer to be expressed on human CD8αβ+ effector-memory cells (1) and expansion of CD8α+CD8β^low^ cells has been described with age (6) and in patients with HIV-1 (7), SLE (8), and Wiskott–Aldrich syndrome (9). CD8α+CD8β^low^ populations have been previously described to be either CD28+ or CD28− (8) and similar expansions have been described in post-chemotherapy patients with Hodgkins disease as a highly differentiated CD57+ perforin+ subset (10). In view of our recent findings of SP CD8αα expression linked to CD161++ MAIT cells, there is a need to more definitively assess human CD8αα expression in diverse human T cell populations in both health and disease.

The CD8αβ co-receptor binds to the MHC class I molecule, stabilizing the interaction between the TCR and the cognate peptide-MHC-I complex (11), triggering T cell activation through intracellular interaction of the CD8α cytoplasmic tail with the Src-family protein kinases Lck and LAT and subsequent phosphorylation of the TCR-CD3 complex (12). CD8 T cells vary by several orders of magnitude in their sensitivity to peptide antigen bound to MHC-I (13, 14). This is determined on the T cell side by the TCR affinity for the peptide-MHC-I complex, the level of TCR expression, TCR valency, accessory/co-stimulatory molecule expression, and CD8 αβ co-receptor expression. CD8αβ co-receptor dependence varies inversely with affinity of the TCR (15–19) and very high-affinity T cells can be activated independent of CD8αβ binding (17). T cell sensitivity is an important factor in the immune control of viral infection (20) and may play a role in outcomes from HIV (21).

Although both CD8αβ and CD8αα bind soluble MHC-I with similar affinity in Biacore experiments (22) and it is the cytoplasmic domain of the CD8α chain which interacts with Lck/LAT, CD8αβ enhances T cell sensitivity to cognate antigen by 100-fold compared to cells only expressing CD8αα (23, 24). It has been suggested that this might be explained by the fact that CD8αα is excluded from lipid rafts. Further to this, data suggests that CD8αα may actively inhibit T cell activation, as co-expression on CD8αα T cells decreases sensitivity to their cognate antigen (25), although the mechanism for this is not known. In mice, induction of expression of CD8αα by high-affinity memory precursors is thought to prevent their activation-induced cell death and exhaustion of chronically activated effector cells as in chronic viral infection (4).

CD8αβ T cells undergo repeated rounds of cell division and differentiation, sequentially acquiring characteristic phenotypic and functional features of early, intermediate, and late differentiation (26, 27). Late-differentiated cells are described as effector-memory cells (CD45RA±, CCR7−) characterized by loss of expression of the co-stimulatory molecules CD28 and CD27 and up-regulation of the senescence marker CD57. They are also found to have altered functional characteristics, with reduced production of IL-2 (and associated proliferative capacity) and increased cytotoxicity and expression of inflammatory cytokines IFN-γ and TNF-α. HIV-1 infection is associated with the development of prematurely senescent immune system; massive activation of the whole CD8 T cell population is observed during acute infection (28) and a large population of CD28−CD27−CD57+ T cells is found within bulk CD8 T cells. In parallel to the negative implications in aged immune systems (with increased susceptibility to infectious diseases and cancer, reduced effectiveness of vaccinations, and increased autoimmunity) (29–32), there is a significant association between the size of the CD28−CD27−CD57+CD8+ T cell population and HIV-1 disease progression (33). A similar observation of a large CD28−CD8 T cell population has also been made in chronic hepatitis B (HBV) associated with higher viral load and liver inflammation (34). Previous descriptions of expanded CD8α+CD8β^low^ populations have independently described them to be CD28−(8) and CD57+ perforin+ subset (10), however there has been no definitive study to date to bring these observations together.

Thymic leukemia antigen is a non-classical MHC class I molecule in mice. X-ray crystallography of the TL structure has demonstrated that its antigen-binding groove is occluded and so does not play any role in antigen presentation (35); however a number of publications have demonstrated that it binds murine CD8αα with much higher affinity than CD8αβ (22, 36–38). No human homolog of TL has been identified to date; however murine TL does bind to human CD8αα molecules with high-affinity (1) and a TL-tetramer can be used to demonstrate expression of CD8αα at the cellular surface of antigen-experienced human CD8αβ T cells.

Here, using both co-CD8α/CD8β antibody staining to detect CD8αβ expression and a TL-tetramer to detect CD8αα expression we definitively describe human CD8αα expressing T cell subsets within both the CD161++ MAIT and CD161+/CD161-non-MAIT CD8+ T cell populations. In addition, we have shown CD161−CD8α+CD8β^low^ T cells to be a late-differentiated population that dominates in chronic infections such as those caused by HBV and HIV-1.

## Materials and Methods

### Study subjects

Sixteen adult healthy controls (HC), 31 patients with chronic HBV, 23 patients with chronic HCV, and 10 patients with HIV-1 infection were enrolled in the study. All study subjects were recruited following informed consent and in agreement with the Oxfordshire Research Ethics Committee. Patient demographics are summarized in Table 1.

**Table 1:** **Demographics table**.

Disease	No.	Sex M:F	Age (years)	Viral load (copies/ml)	ALT (U/ml)	e-Antigen status ±	CD4 count (cells/mm^3^)	CMV status ±
HBV	31	24:7	37 (21–64)	1.69 × 10^8^ (0–1.7 × 10^8^)	69 (12–881)	6/25	NA	20/3
HCV	23	11:12	47 (28–67)	2.25 × 10^6^ (2.8 × 10^4^–1.8 × 10^7^)	52 (21–150)	NA	NA	13/7
Treated HBV	10	6:4	40 (27–49)	27 (0–100)	31 (13–63)	6/4	NA	ND
HIV-1	10	ND	ND	4.8 × 10^4^ (758–1.9 × 10^5^)	NA	NA	456 (51–1080)	10/0

*NA, not applicable; ND, no data*.

### T cell recovery

Peripheral blood mononuclear cells (PBMCs) were isolated from EDTA peripheral blood samples by Ficoll-Histopaque density gradient centrifugation (Lymphoprep, Axis Shield). Samples not used for immediate analysis were then frozen in a mix of DMSO (25%), RPMI media (25%) (GibcoBRL), and fetal calf serum (50%) and stored in liquid nitrogen prior to subsequent analysis.

### Antibodies

Anti-CD8α-PerCP, anti-CD3-AmCyan, anti-CD3-FITC, anti-CD8β-APC, anti-CD4-Alexa 700, anti-IFN-γ-Alexa 700, anti-TNF-α-PeCy7, anti-IL-2-FITC, anti-Ki67-FITC, anti-CD62L-PeCy7, anti-CD45RA-FITC and anti-CD62L-APC, anti-CCR7 PeCy7 (BD); Anti-CD3-Pacific orange and live/dead violet fixable cell stain (Invitrogen); anti-CB8β-PE and anti-CD161-PE (Beckman Coulter); anti-CD161-APC (Miltenyi).

### Immunofluorescent staining

Cryopreserved PBMCs were incubated with anti-surface antigen antibodies at 4°C for 20 min, washed in phosphate buffered saline (PBS) and fixed with 1% formaldehyde in PBS. Intracellular cytokine staining was performed on cryopreserved PBMCs in complete medium (RPMI 1640 containing 10% FCS, 1% streptomycin/penicillin, and l-Glutamine), stimulated with leukocyte activation cocktail (BD) and incubated (37°C, 5% CO_2_) or 4 h. Cells were washed in PBS and stained with antibodies against surface antigens and incubated at 4°C for 20 min. After fixation/permeabilization (FoxP3 staining kit, BD), cells were stained with antibodies against intracellular antigens, incubated at 4°C for 20 min and washed in 10% PermWash buffer (BD) in sterile de-ionized water.

### HIV-1 tetramer synthesis and staining

HLA-B^∗^42:01 heavy chain was expressed in Rosetta (DE3)pLys (Novagen), purified, and refolded around the peptide of interest in the presence of human β2M light chain. Unrefolded heavy chain and peptide were separated from refolded MHC:peptide monomer complexes using FPLC prior to tetramerization of monomers and conjugate ion to R-phycoerythrin (Extravidin PE, Sigma) to obtain PE labeled HLA-B^∗^42:01 tetramers.

Antigen-specific CD8 T cell responses were studied in HLA-B^∗^42:01+ HIV-1+ patients from the cohort described in Table 2. Streptavidin-PE conjugated B42:01 tetramers were incubated with thawed patient PBMCs at a concentration of 1 in 10 for 20 min at room temperature. Samples were then washed in PBS and stained with a panel of antibodies against surface antigens for 20 min at 4°C. Samples were then washed in PBS and fixed in 1% formaldehyde prior to FACS analysis.

**Table 2:** HIV-1 derived epitopes presented by HLA-B*42:01 alleles.

Protein	Epitope name	HXB2 location	Amino acid sequence
p24	p24-TL9	P24-Gag180–188	TPQDLNTM L
Int	Int-IM9	Int-Pol28–36	IIKDYGKQ M
Nef	Nef-RM9	Nef70–78	RPQVPLRP M
	Nef-TL10	Nef128–137	TPGPGVRYPL
Vpr	Vpr-FL9	Vpr34–75	FPRPWLHG L
Vif	Vif-HI10	Vif48–57	HPKVSSEVHI

### TL-tetramer staining

Thymic leukemia monomers, produced as previously described (22) were tetramerized using streptavidin-PE (Molecular probes). PBMCs from both patients and HCs were pre-incubated for 15 min at 23°C with unlabeled anti-CD8α (2.5 μg/ml) (SK1; BD) or anti-CD8β (25 μg/ml) (2ST8.5H7; BD). After washing in PBS TL-tetramer (1:100) was incubated for 10 min at 23°C. PBMCs were then stained with a panel of antibodies (as above) to surface antigens for 20 min at 4°C and subsequently washed in PBS and fixed in 1% formaldehyde prior to FACS analysis.

### CMV immunoglobulin G ELISA

Qualitative ELISA was performed on cryopreserved patient serum samples using the Diamedix MCV IgG kit. 1:101 dilutions of serum where made using sample diluent. Hundred microliters of standards, controls, and prepared diluted patient samples were added to the antigen wells and incubated at 37°C for 60 min. Conjugate was then added to each sample and incubated for a further 60 min at 37°C. The wells were then washed and 100 μl of substrate solution added to each well and incubated for a further 20 min at 37°C. Hundred microliters of stop solution was then added and the wells read by a plate reader at 450 nm.

### Flow cytometry

All samples prepared for FACS analysis were acquired within 24 h of staining on an LSR II cytometer using FACS diva software (BD). Data was subsequently analyzed using FlowJo software (Tree star). Gating was defined using a “Full minus one” strategy.

### Statistical analysis

Statistical analysis was performed using GraphPad PRISM (GraphPad software) version 4. A *p* value of<0.05 was considered significant.

## Results

### Defining CD8β expression levels by CD161++, CD161+, and CD161− CD8α+ T cell subsets

**Figure 1:**
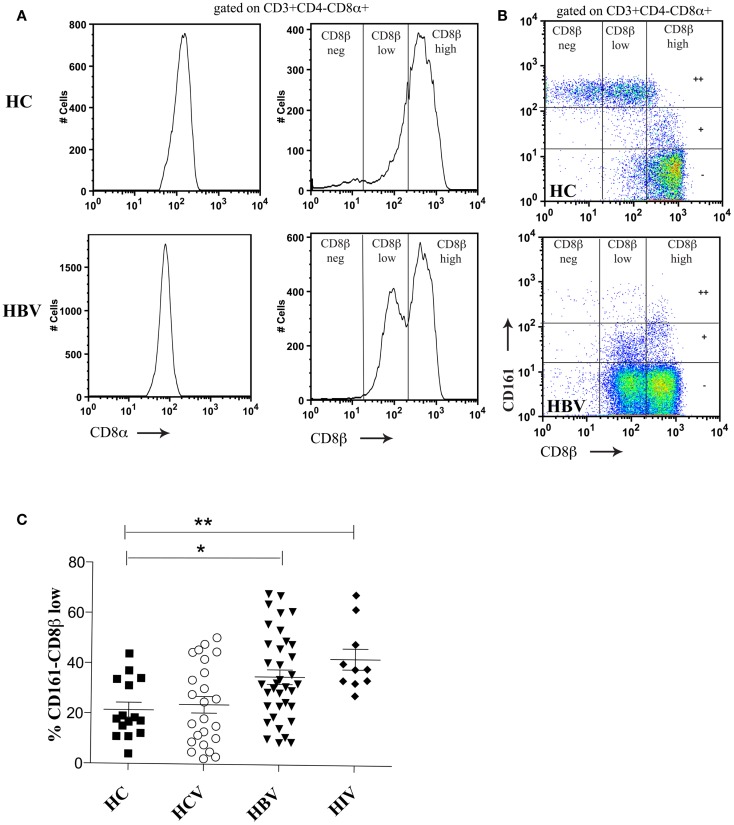
**Distinct CD8β expression levels are demonstrated on CD161++, CD161+, and CD161−CD8α+ T cell subsets**. **(A)** Co-staining of PBMCs with anti-CD8α and anti-CD8β antibodies identifies a range of CD8β expression by CD3+CD4−CD8α+ T cells in both a HC and patient with HBV. **(B)** Distinct CD8β^high^, CD8β^low^, and CD8β^neg^ populations are demonstrated based on the level of CD161 expression (marked on the vertical gates on FACS plots as++/±). Representative FACS plots of both HC (*n* = 16) and patients with chronic HBV (*n* = 31) shown. **(C)** The size of the CD161−CD8α+ CD8β^low^ T cell population as a percentage of the CD161−CD8α+ population is compared between HCs and patients with chronic HBV, HCV, and HIV-1 infections. (*p < 0.05, **p < 0.001, One-way ANOVAs).

We first addressed the distribution of CD8β staining in peripheral blood; since we have previously shown a substantial impact of CD161 expression on this, we analyzed the CD161++, CD161+, and CD161− T cell populations in parallel. T cells expressing CD8αα were identified using a dual staining strategy with anti-CD8α and anti-CD8β fluorochrome-labeled antibodies for FACS analysis. As shown in both HC blood and during HBV infection (Figure 1A), there is a broad range of CD8β expression by the CD3+CD4−CD8α+ population in peripheral blood in the context of a single peak of CD8α expression. Further to this, using anti-CD161 co-staining it is possible to identify three distinct populations of CD3+CD4−CD8α+ T cells. The SP CD8αα (CD8α+CD8β^neg^) population is exclusive to the CD161++ MAIT cell population as demonstrated previously (5) and in the HC shown (Figure 1B). The CD161++ population was noted to be significantly reduced in chronic HBV, as previously described (39, 40).

Within the CD161+ and CD161− populations it was possible to identify two CD3+CD4−CD8α+ populations based on CD8β expression (CD8β^high^ and CD8β^low^). The CD161−CD8α+CD8β^low^ population was significantly expanded in patients with chronic HBV and HIV-1 compared to HCs (Figure 1C). Importantly, CD161−CD8α+CD8β^low^ T cells expressed an αβ and not a γδ TCR (data not shown). The clone of CD8β antibody used (2ST8.5H7) binds to an epitope on CD8β which requires expression of both the CD8α and CD8β chains. Co-staining with a separate CD8α antibody allows for identification of populations with maintained CD8α but reduced CD8αβ expression. By this indirect method, we conclude that the CD8α+CD8β^low^ population expresses a mixture of CD8αα and CD8αβ molecules on the cell surface due to relative levels in CD8α and CD8β expression.

**Figure 2:**
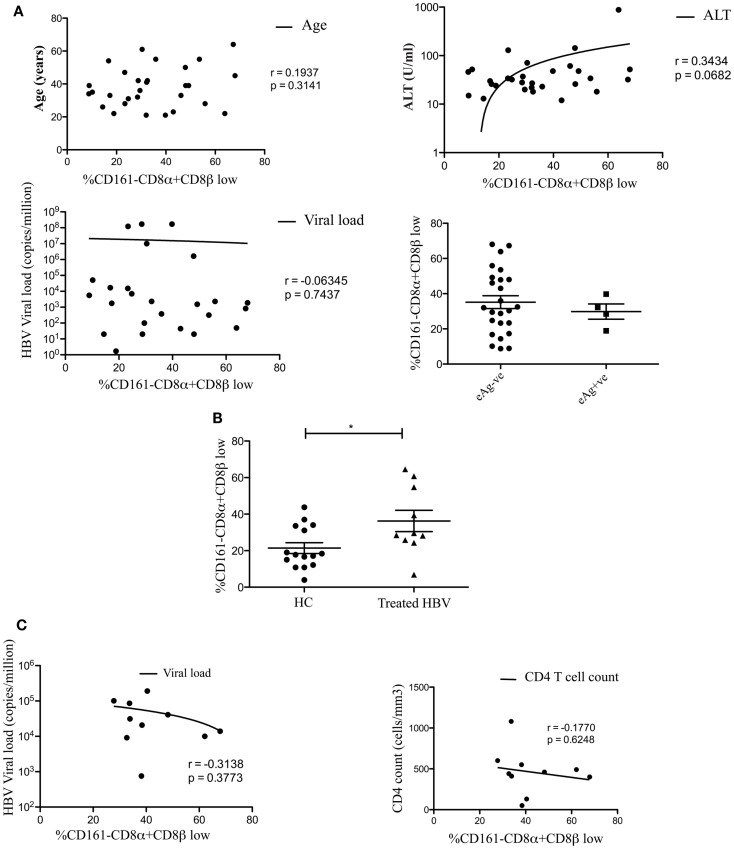
**CD161−CD8α+CD8β− T cell populations develop independent of clinical status in chronic HBV and HIV-1 infections**. **(A)** Analysis of the correlation between the size of the CD161−CD8α+CD8β^low^ T cell population and patient age/viral load (Pearson) and comparison of the size of the CD161−CD8α+CD8β^low^ population, as a proportion of the CD161−CD8α+ population, between patients e-antigen positive and e-antigen negative patients (Mann Whitney test) in chronic HBV infection. **(B)** Analysis of the % of CD161−CD8α+CD8βlow T cells as a proportion of the CD161−CD8α+ population in patients with treated with chronic HBV compared to HC (*p < 0.05, unpaired *t* test). **(C)** Correlative analysis between the size of the CD161−CD8α+CD8β^low^ T cell population as a proportion of the CD161−CD8α+ population and viral load/CD4 count in HIV-1 infection (Pearson).

**Figure 3:**
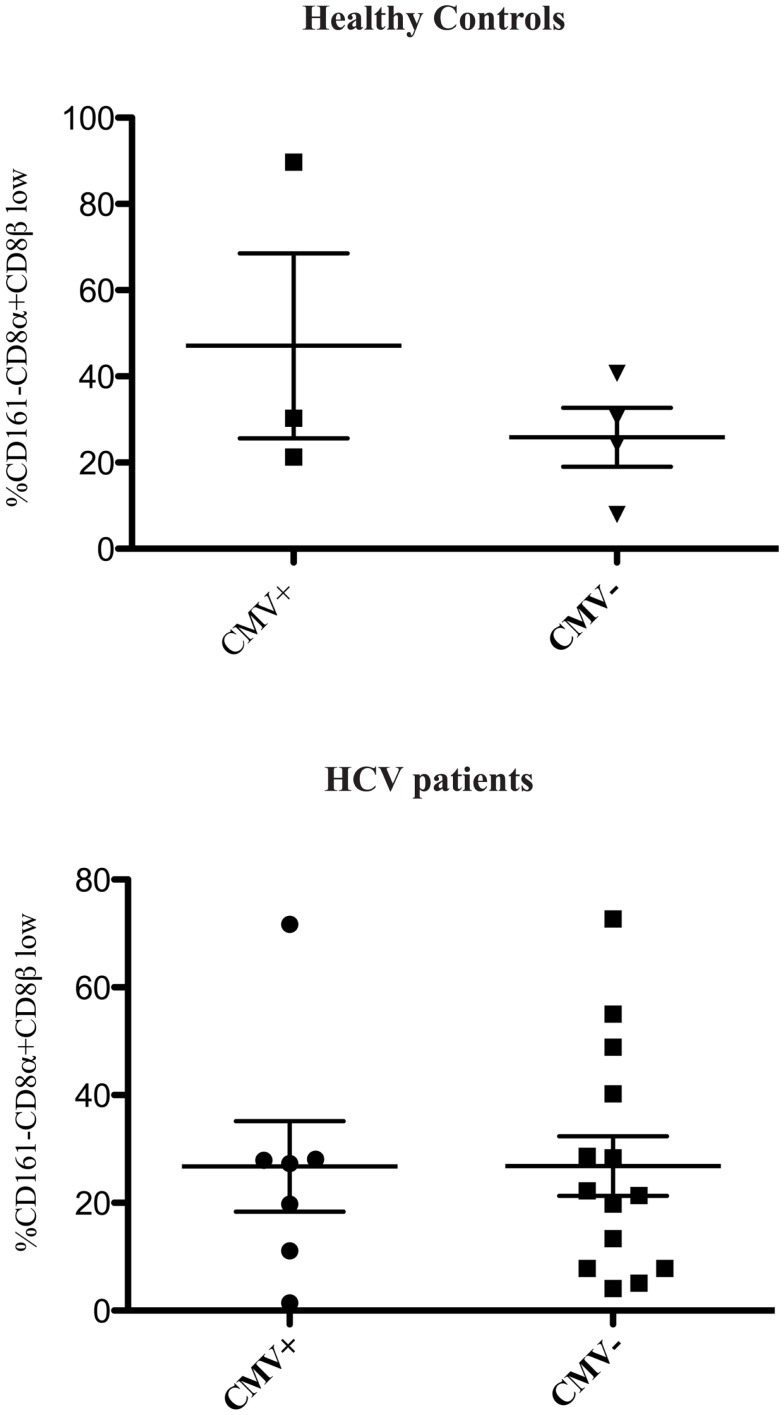
**CD161−CD8α+CD8β^low^ population is not related to CMV status**. Cumulative data comparing the % of CD161−CD8α+CD8β low cells as a proportion of CD161−CD8α+ T cells in healthy controls and patients with HCV based on CMV status (Mann Whitney test).

We found no association between the proportion of CD161−CD8α+CD8β^low^ T cells and disease activity in either chronic HBV or HIV-1 infections (Figure 2). Additionally we did not find an association between the proportion of CD161−CD8α+CD8β^low^ T cells and CMV seropositivity, so we were able to exclude this as responsible for the differences in CD161−CD8α+CD8β^low^ populations observed in diseased vs. healthy cohorts (Figure 3).

### The CD161−CD8α+CD8β^low^ T cell population expresses high levels of CD8αα

**Figure 4:**
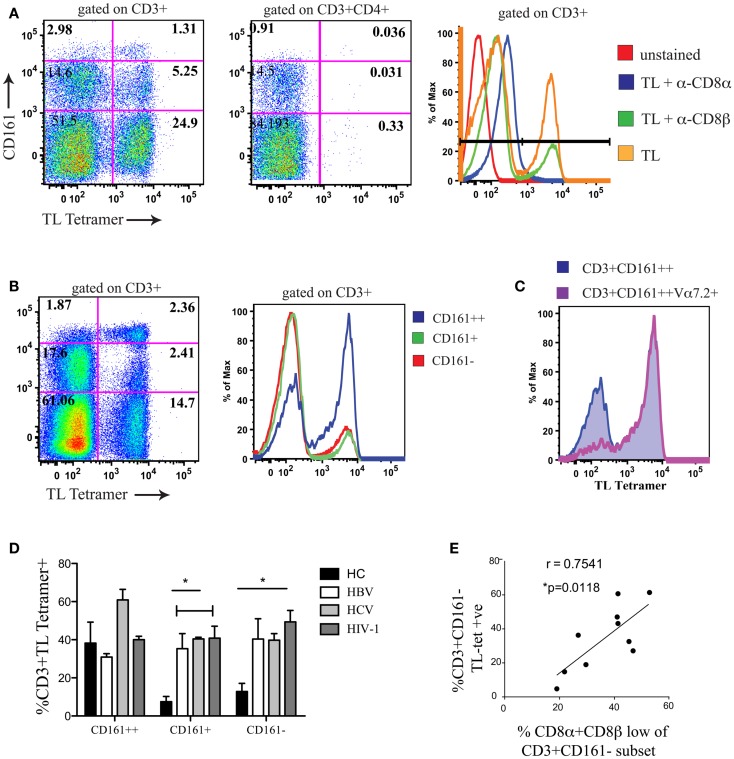
**A positive correlation is observed between CD8αα expression and the size of the CD8α+CD8β^low^ population**. **(A)** Representative FACS data showing TL-tetramer binding to CD3+ and CD3+CD4+ T cells of peripheral blood lymphocytes. CD8αβ binding by TL-tetramer is blocked by α-CD8β antibody clone 2ST8.5H7. Control samples shown on histogram on right (Negative controls: unstained and TL + α − CD8α; Positive control; TL). Data shown from a patient with chronic HBV. Data representative of 11 repeat experiments. **(B)** Representative FACS data using TL-tetramer staining to demonstrate CD8αα expression by CD3+CD161++/CD161+/CD161− T cells in HCs. Data representative of three repeat experiments. **(C)** Representative FACS data of TL-tetramer staining/CD8αα expression by CD3+CD161++ and CD3+CD161++Vα7.2+ T cell subsets in a HC. Data representative to three repeat experiments. **(D)** Comparison of TL-tetramer expression/CD8αα expression between CD161++/CD161+/CD161−T cell subsets in HCs and patients with chronic HBV, HCV, and HIV-1 infections. (*p < 0.05, One-way ANOVAs). **(E)** Correlative analysis between the proportion of CD3+CD161− TL-tetramer positive cells and the % of CD161−CD8β^low^ cells as a proportion of the CD161−CD8α+ population in patients with chronic HBV, HCV, and HIV-1 infections, *r* = 0.7541, *p* = 0.0118, Pearson.

To confirm that CD161−CD8α+CD8β^low^ T cells co-expressed CD8αα, we used a TL-tetramer which binds to both CD8αα and CD8αβ; this demonstrated, as previously, that following CD8αβ blockade using the anti-CD8β antibody clone 2ST8.5H7 and an anti-CD8α antibody as a negative control to completely block CD8αα binding, TL-tetramer staining is specific to the CD8αα population in humans (Figure 4A) (1). Previous experiments have validated this method to identify CD8αα and CD8αβ co-expressing cells using dual staining with a labeled CD8β antibody and TL-tetramer (22, 41).

We subsequently demonstrated, using the TL-tetramer, that in HC CD8αα is highly expressed on the CD161++ MAIT cell subset of PBMCs (Figure 4B). This is in keeping with our previous data using dual CD8α and β staining which found the SP CD8αα (CD8α+CD8β^neg^) population in humans to be restricted to this subset (5). In addition we observed that within the CD161++ CD8+ population, CD8αα was almost exclusively expressed by the Vα7.2+CD161++ T cells (Figure 4C).

Using the TL-tetramer, we further investigated CD8αα expression by the CD161−CD8α+CD8β^low^ population in patients with chronic viral infection. In comparison with HC, where CD8αα is expressed mainly by the CD161++ cells, in patients with chronic HBV, HCV, and HIV-1 we found a significant number of TL-tetramer positive cells among the CD161+ and CD161− non-MAIT CD8+ T cell populations (Figure 4D). Furthermore, we demonstrated a clear correlation between the proportion of CD161−CD8β^low^ T cells and the TL-tetramer+ T cells in these chronically infected patients (Figure 4E) indicating that the CD8α+CD8β^low^ population identifies a population of CD8αα-expressing CD8αβ T cells.

### CD8αα-expressing CD161−CD8α+CD8β^low^ T cells are an effector-memory population

**Figure 5:**
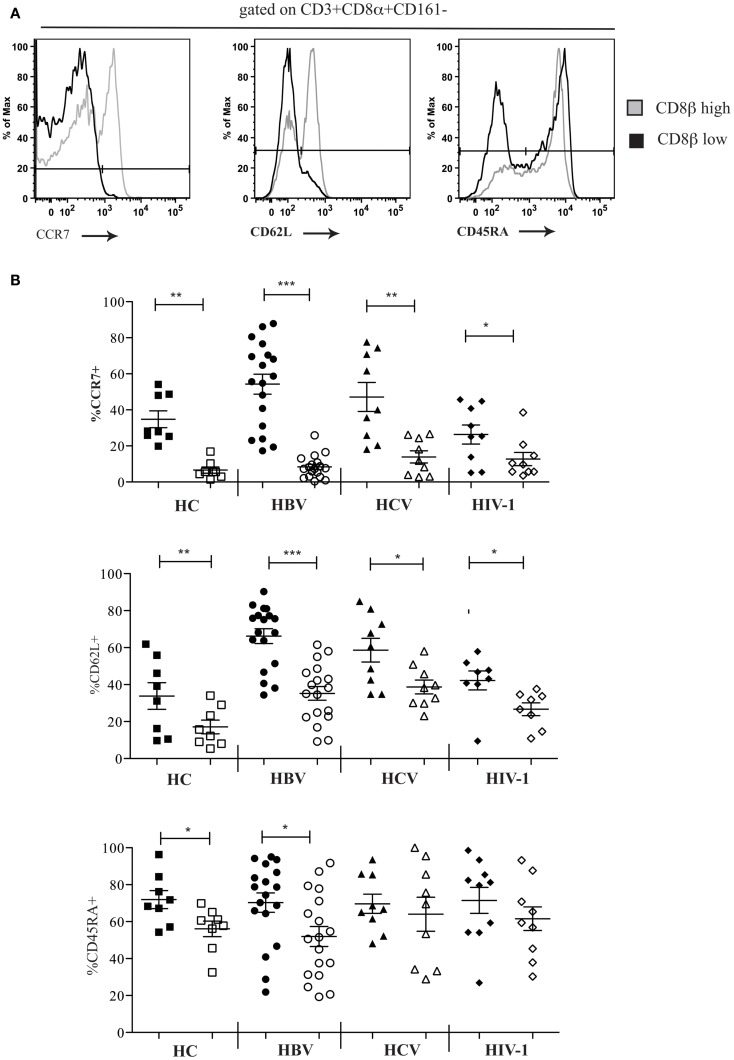
**CD161−CD8α+ T cells are an effector-memory population**. **(A)** Representative FACS data comparing CCR7, CD62L, and CD54RA expression by peripheral blood CD161−CD8α+CD8β^low^ and CD161−CD8α+CD8β^high^ T cell populations as demonstrated here by a patient with chronic HBV infection. **(B)** Cumulative data comparing CCR7, CD62L, and CD54RA expression by peripheral blood CD161−CD8α+CD8β^low^ and CD161−CD8α+CD8β^high^ T cell populations in HCs and patients with chronic HBV, HCV, and HIV-1 infections. CD161−CD8α+CD8β^high^ T cell and CD161−CD8α+CD8β^low^ T cells represented on graphs by filled and unfilled shapes respectively. (*p < 0.05, **p < 0.001, ***p < 0.0001, Wilcoxon signed rank test).

We next characterized the phenotype of the CD161−CD8α+CD8β^low^ population. Using FACS analysis we were able to show that in comparison the CD161−CD8α+CD8β^high^ population, CD161−CD8α+CD8β^low^ T cells were mostly CCR7− and CD62L− in the peripheral blood of both HC and patients with chronic HBV, HCV, and HIV-1 infections (Figures 5A,B). In HC and in chronic HBV infection there was significantly lower expression of CD45RA by CD161−CD8α+CD8β^low^ compared to CD161−CD8α+CD8β^high^ T cells, but significance was not met in the same comparison in patients with chronic HCV and HIV-1 infections, indicating that they may have re-expressed CD45RA – a recognized phenomenon of late-differentiated cells (42).

### CD8αα-expressing CD161−CD8α+CD8β^low^ T cells express markers of late differentiation and activation

**Figure 6:**
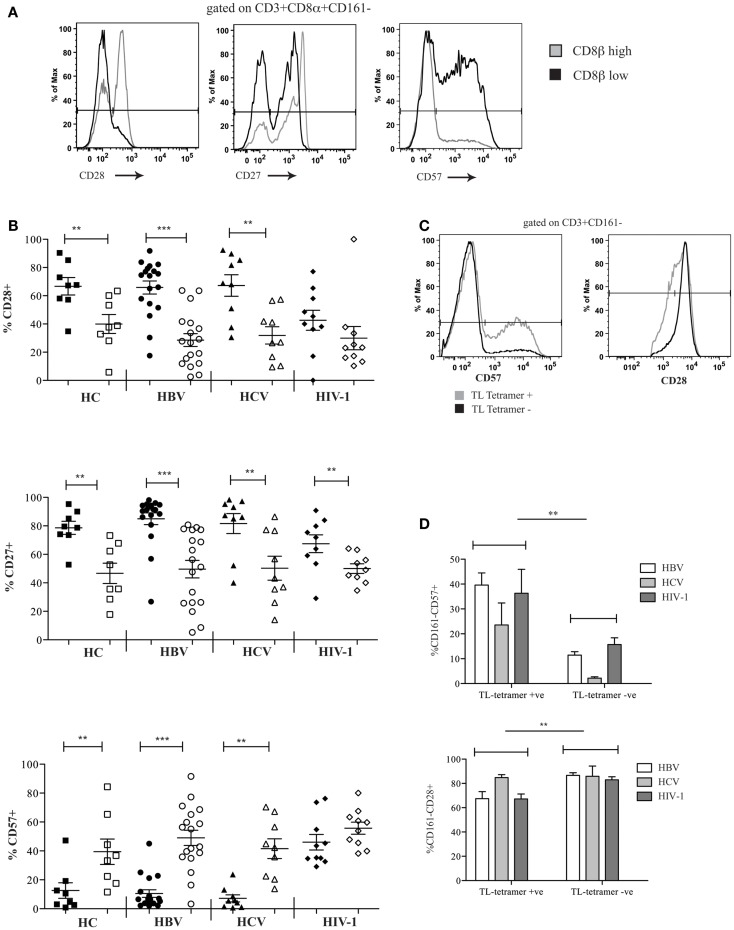
**CD161−CD8α+CD8β^low^ T cells express markers of late differentiation**. **(A)** Representative FACS data comparing CD28, CD27, and CD57 expression by peripheral blood CD161−CD8α+CD8β^low^ and CD161−CD8α+CD8β^high^ T cell populations as demonstrated by a patient with chronic HBV infection. **(B)** Cumulative data comparing CD28, CD27, and CD57 expression by peripheral blood CD161−CD8α+CD8β^low^ and CD161−CD8α+CD8β^high^ T cell populations in HCs and patients with chronic HBV, HCV, and HIV-1 infections. CD161−CD8α+CD8β^high^ T cell and CD161−CD8α+CD8β^low^ T cells represented on graphs by filled and unfilled shapes respectively. (**p < 0.001, ***p < 0.0001, Wilcoxon signed rank test). **(C)** Representative FACS data comparing CD57 and CD28 expression between CD3+CD161− TL-tetramer+ and CD3−CD161− TL-tetramer – cells in a patient with chronic HBV. **(D)** Cumulative data comparing CD57 and CD28 expression between CD3+CD161− TL-tetramer+ and CD3+CD161− TL-tetramer – cells for patients with HBV, HCV, and HIV-1 infections (**p < 0.001, Wilcoxon signed rank test).

We investigated the differentiation state of the CD8αα-expressing, CD161−CD8α+CD8β^low^ peripheral blood T cell population and demonstrated significantly reduced expression of CD28, CD27, and increased CD57 in HC and patients with chronic HBV and HCV compared to the CD161−CD8α+CD8β^high^ T cells. In HIV-1 infected patients, we saw a significant difference only in the expression of CD27 between the CD161−CD8α+CD8β^low^ and CD161−CD8α+CD8β^high^ T cells (Figures 6A,B). Overall this may be explained by increased expression of CD57 and decreased expression of CD28 by both the CD161−CD8α+CD8β^low^ and the CD161−CD8α+CD8β^high^ subsets. Consistent with this, CD57 expression was significantly higher in both subsets in HIV-1 patients compared to the HC (*p* < 0.05, one-way ANOVA).

To confirm these findings with the TL-tetramer, we repeated the experiments using the staining protocols described above to identify CD8αα-expressing cells. These experiments showed that TL-tetramer+ cells in patients with chronic HBV and HIV-1 had significantly increased CD57 expression and decreased CD28 expression, in keeping with the observation that CD161−CD8α+CD8β^low^ T cells express high levels of CD8αα (Figures 6C,D).

**Figure 7:**
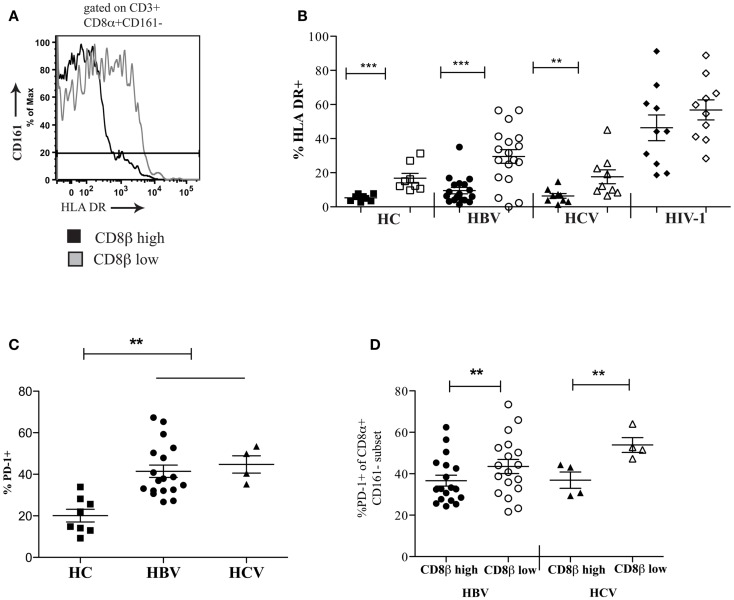
**CD161−CD8α+CD8βlow T cells express markers of activation and exhaustion**. **(A)** Representative FACS data comparing HLA-DR expression between peripheral blood CD161−CD8α+CD8β^low^ and CD161−CD8α+CD8β^high^ T cell populations. **(B)** Cumulative data comparing HLA-DR expression between CD161−CD8α+CD8β^low^ and CD161−CD8α+CD8β^high^ T cell populations in HCs and patients with chronic HBV and HCV. CD161−CD8α+CD8β^high^ T cell and CD161−CD8α+CD8β^low^ T cells represented on graphs by filled and unfilled shapes respectively. (**p < 0.001, ***p < 0.0001, Wilcoxon signed rank test). **(C)** Comparison of PD-1 expression by CD8 T cells in patients with chronic HBV and HCV infections compared to HCs (**p < 0.001, One-way ANOVA). **(D)** Comparison of PD-1 expression by CD161−CD8α+CD8β^low^ and CD161−CD8α+CD8β^high^ T cell populations in chronic HBV and HCV (**p < 0.001, Wilcoxon signed rank test).

To extend these findings, we addressed whether CD8αα-expressing T cells also showed other markers of activation or exhaustion. We studied a panel of activation markers (CD69, CD25, CD38, HLA-DR) and found the late activation marker HLA-DR to be significantly up regulated on the CD161−CD8α+CD8β^low^ T cell population compared to the CD161−CD8α+CD8β^high^ T cell subset (Figures 7A,B). In HIV-1 infected patients the difference between the CD161−CD8α+CD8β^low^ and CD161−CD8α+CD8β^high^ T cell populations appeared to be abolished due to up-regulation of HLA-DR on both subsets compared to the HC CD161−CD8α+CD8β^high^ population (*p* < 0.0001, one-way ANOVA). We also found that overall there was increased expression of the exhaustion marker PD-1 by CD161−CD8+ T cells in patients with chronic HBV and HCV compared to HC (Figure 7C) and, importantly, consistently greater expression by the CD161−CD8α+CD8β^low^ compared to the CD161−CD8α+CD8β^high^ T population in the patient groups studied (Figure 7D).

### CD8αα-expressing CD161−CD8α+CD8β^low^ T cells are functionally distinct from CD161−CD8α+CD8β^high^ T cells

**Figure 8:**
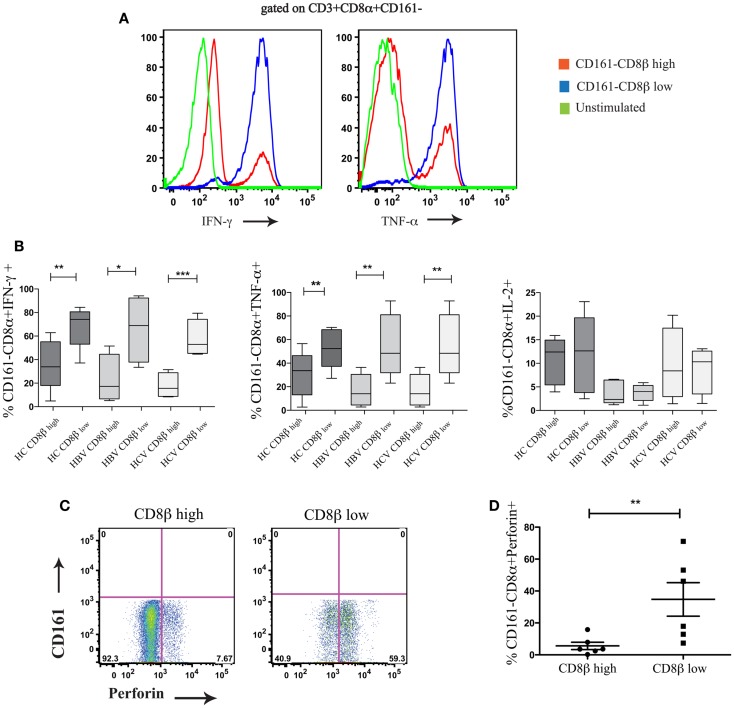
**CD161−CD8α+CD8β^low^ T cells are functionally distinct from CD161−CD8α+CD8β^high^ T cells**. **(A)** Representative FACS data comparing IFN-γ and TNF-α production by CD161−CD8α+CD8β^low^ and CD161−CD8α+CD8β^high^ T cell populations following stimulation with PMA and Ionomycin. **(B)** Cumulative data comparing percentages of IFN-γ, TNF-α, and IL-2 positive cells within the CD161−CD8α+CD8β^low^ and CD161−CD8α+CD8β^high^ T cell populations in chronic HCV and HBV (**p < 0.001, ***p < 0.0001 paired *t* test). **(C)** Representative FACS plots comparing perforin expression between CD161−CD8α+CD8β^low^ and CD161−CD8α+CD8β^high^ T cells. **(D)** Cumulative data comparing levels of perforin expression between CD161−CD8α+CD8β^low^ and CD161−CD8α+CD8β^high^ T cells in HCs and patients with chronic HBV showing CD161−CD8α+ CD8β^low^ (**p < 0.001, ***p < 0.0001, Wilcoxon ranked test).

We next compared the functionality of CD8αα-expressing CD161−CD8α+CD8β^low^ and CD161−CD8α+ CD8β^high^ T cell subsets. By stimulating whole PBMCs from HC and patients with chronic HBV and HCV with PMA/Ionomycin we found that there were significant functional differences between these populations. The CD8αα-expressing CD161−CD8α+CD8β^low^ T cell population produced significantly greater amounts of IFN-γ and TNF-α compared to CD161−CD8α+CD8β^high^ T cell population across HC and the patient groups studied, although no difference in IL-2 production between the two populations was identified (Figures 8A,B) (i.e., a greater number of cells were cytokine positive, it appears that on a per cell basis – based on MFI, there is no difference between the two populations). In addition, more CD161−CD8α+CD8β^low^ cells expressed perforin compared to their CD161−CD8α+CD8β^high^ counterparts (Figures 8C,D). Overall these data indicate that CD8αα-expressing CD161−CD8α+CD8β^low^ cells have enhanced pro-inflammatory functionality and greater cytotoxicity compared to the CD161−CD8α+CD8β^high^ population; this is in keeping with other published data on the functionality of CD28−CD27−CD57+ CD8+ T cells (43).

### Antigen-specific CD8+ T cells in HIV-1 are found within both CD161−CD8α+CD8β^high^ and CD8β^low^ populations

**Figure 9:**
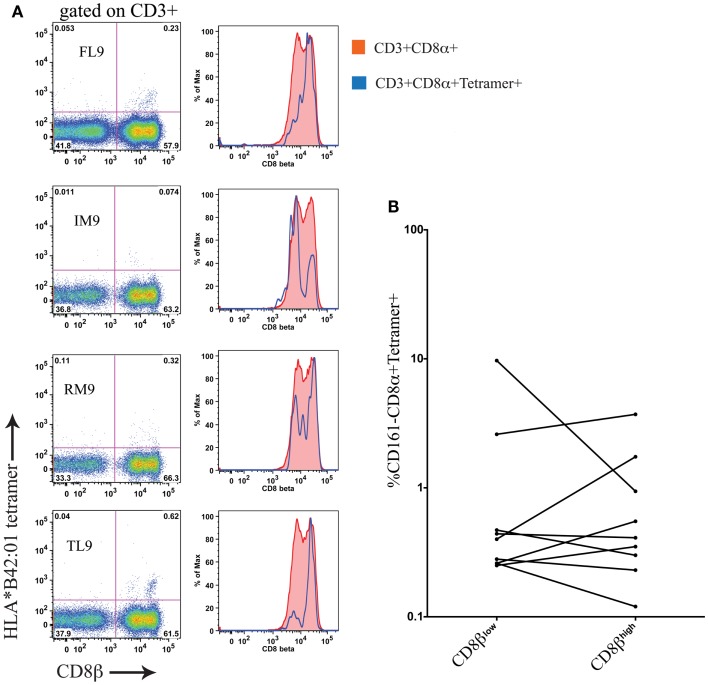
**Antigen-specific CD8 T cells in HIV-1 are CD161−CD8α+CD8β^high^ and CD161−CD8α+CD8β^low^**. **(A)** HLA-B*42:01 tetramer positive CD8 T cell responses in a individual patient with HIV-1 demonstrates antigen-specific T cells are found in both the CD161−CD8α+CD8β^low^ and CD161−CD8α+CD8β^high^ populations of peripheral blood. **(B)** Cumulative data showing distribution of tetramer positive cells between CD161−CD8α+CD8β^high^ and CD161−CD8α+CD8β^low^ T cells in patients. Data from five different patients with responses to epitopes p24-TL9, Int-IM9, Nef-RM9, and Vpr-FL9 are shown.

Using cryopreserved PBMC samples from patients with HIV-1 carrying the HLA-B^∗^42:01 allele we investigated whether antigen-specific CD8 T cells were found within both the CD161−CD8α+CD8β^high^ and CD161−CD8α+CD8β^low^ populations using HLA-B^∗^42:01-peptide tetramers (see Table 2 for details of epitopes). In studying the distribution of antigen-specific responses between the CD161−CD8α+CD8β^high^ and CD161−CD8α+CD8β^low^ populations within an individual we were able to distinguish a variable distribution of the tetramer positive cells, despite an almost 50:50 split of the bulk CD8 population between the CD161−CD8α+CD8β^low^ and CD161−CD8α+CD8β^high^ subsets (Figure 9A). Cumulative data from the antigen-specific populations within the five patients studied using a panel of HLA^∗^B42:01 tetramers (Table 2) demonstrated that overall there is no consistent bias toward either the CD161−CD8α+CD8β^high^ or CD161−CD8α+CD8β^low^ populations (Figure 9B).

## Discussion

We have clearly defined human CD8αα-expressing T cell populations using both anti-CD8α/anti-CD8β co-staining and a TL-tetramer within both MAIT and non-MAIT populations. The TL-tetramer used is clearly able to bind CD8αα on CD8β negative cells but can also detect CD8αα on cells expressing CD8β, as previously described (1, 22, 41). In this study, as previously, we blocked CD8β binding throughout to allow for specific detection of CD8αα staining on CD8β^low^ cells (1). Within the MAIT cell population, we have shown CD8αα expression to be most prominent on the Vα7.2 expressing cells of this subset. Further to this we have demonstrated CD8αα expression on both CD161+ and CD161− subsets. We have shown CD8αα-expressing CD161−CD8αβ (CD161−CD8α+CD8β^low^) T cells are significantly increased in patients with chronic HBV and HIV-1 infections compared to HC and have brought together previous data to definitively demonstrate expression of CD8αα on such cells is associated with terminally differentiated effector-memory T cells (CCR7−CD62L−CD45RA−CD28−CD27−CD57+). Consistent with activation-induced expression of CD8αα, these T cells also express HLA-DR.

Although the CD161−CD8α+CD8β^low^ T cells are also present in HC, it is important to consider what might be driving the significant expansion of these cells in the context of chronic HBV and HIV-1 infections. In HIV infection, we found no association with the proportion of CD161−CD8α+CD8β^low^ T cells and viral load or CD4 count. Equally in HBV infection, there was no association with liver inflammation (as reflected by ALT), e-antigen status, or age of the patient. Thus the overall relationship between the populations and pathogenesis is not yet defined. There are two possible suggestions. Firstly, the expansion of the CD8αα-expressing T cells could be driven by inflammatory cytokines or disease activity. This may explain the very large magnitude of the effect, which is affecting a set of cells likely much greater than the size of the HBV- or HIV-specific CD8+ T cell population. The lack of clear association with disease status is not consistent with a direct causal impact, but might be consistent with widespread changes in T cell phenotype reported previously (28, 33). Alternatively, the expression of CD8αα might be linked to TCR-driven T cell maturation. This would explain the close links seen with expression of other phenotypic markers, and the known regulation previously determined in mouse models. Whereas CD8αβ TCR co-receptor is constitutively expressed on naïve T cells, CD8αα is transiently induced on strongly activated CD8αβ effector T cells (4) and its expression can be maintained on chronically activated memory CD8 T cells, driven by IL-15 (44, 45). Potentially therefore, a combination of antigen-specific and “off-target” effects could explain the findings in HBV and HIV seen here, although the differences between HCV and these diseases still requires further exploration.

Regardless of the underlying stimulus mediating up-regulation of CD8αα on T cells, what is its impact once expressed? In contrast to CD8αβ, CD8αα functions as a TCR repressor and co-expression of CD8αα on activated cells coincides with an increased threshold for activation (25, 46). Consequently, CD8αα expressed on chronically activated CD8+ T cells might prevent exhaustion of these cells or reduce the risk for excessive or aberrant cytolytic responses. Thus CD8αα may act like other “tuning” molecules to aid survival, in a manner analogous to expression of KIRs or other NK cell associated receptors on T cells (42). Indeed, previous phenotyping of CD8β^low^ cells would suggest this to be the case with increased expression of CD161, CD16, CD158α, and NKB1, although high CD161 expression on CD28+CD8β^low^ T cells likely describes the MAIT cell population (47) and emphasizes the need for this population to be considered when analyzing CD8αα-expressing T cells.

Similar accumulation of CD8αα-expressing CD27−CD28−CD57+CD8+ memory T cells would be predicted in the elderly and also in patients with autoimmune conditions (29–32). The consequences of this on T cell sensitivity requires further study and may reveal unique consequences, including marked “off-target” effects for these cells in controlling chronic infections such as HIV-1 and HBV.

## Author Contributions

L. J. Walker, E. Marrinan, J. Ferguson, and M. Muenchhoff conducted the experiments. L. J. Walker and Paul Klenerman designed the study. H. Kloverpris, M. Muenchhoff, P. Goulder, E. Barnes, and H. Cheroutre provided samples and reagents used in the study. L. J. Walker and Paul Klenerman wrote the paper. H. Cheroutre read and provided comments on the paper.

## Conflict of Interest Statement

The authors declare that the research was conducted in the absence of any commercial or financial relationships that could be construed as a potential conflict of interest.
